# Phantom Tumor of the Lung: Localized Interlobar Effusion in Congestive Heart Failure

**DOI:** 10.1155/2014/207294

**Published:** 2014-09-28

**Authors:** Mislav Lozo, Emilija Lozo Vukovac, Zeljko Ivancevic, Ivan Pletikosic

**Affiliations:** ^1^Division of Cardiology, Department of Internal Medicine, University Hospital Centre Split, Spinciceva 1, 21000 Split, Croatia; ^2^Department of Pulmonary Diseases, University Hospital Centre Split, Spinciceva 1, 21000 Split, Croatia; ^3^University of Split School of Medicine, Soltanska 2, 21000 Split, Croatia

## Abstract

Localized interlobar effusions in congestive heart failure (phantom or vanishing lung tumor/s) is/are uncommon but well known entities. An 83-year-old man presented with shortness of breath, swollen legs, and dry cough enduring five days. Chest-X-ray (CXR) revealed massive sharply demarked round/oval homogeneous dense shadow 10 × 7 cm in size in the right inferior lobe. The treatment with the loop diuretics and fluid intake reduction resulted in complete resolution of the observed round/oval tumor-like image on the control CXR three days later. Radiologic appearance of such a mass-like configuration in patients with congestive heart failure demands correction of the underlying heart condition before further diagnostic investigation is performed to avoid unnecessary, expensive, and possibly harmful diagnostic and treatment errors.

## 1. Introduction

Phantom or vanishing tumor stands for a localized transudative interlobar pleural fluid collection in congestive heart failure. The name originates from its frequent resemblance to a tumor on the CXR and from its tendency to vanish after appropriate management of heart failure.

## 2. Case Report

An 83-year-old man presented with shortness of breath, swollen legs, and dry cough enduring five days. He had a history of diabetes mellitus type II, hypertension, atrial fibrillation, and congestive heart failure and was treated with insulin, methyldigoxin, furosemide, and peroral potassium supplements. Fine, moist, and subcrepitant inspirations were heard at the left base and diminished breath sounds were present at the right base. The respiratory rate was 32/min. His heart rate was 99/min and his arterial blood pressure was 165/105 mm Hg, and he had a systolic murmur 3/6 over aortic valve projection. Arterial blood gas sampling revealed pH = 7.448, pCO_2_ = 4.33 kPa, pO_2_ = 7.67 kPa, HCO_3_ = 22.0 mmol/L, base excess −1.1 mmol/L, and SaO_2_ = 91.4%. Laboratory studies were remarkable for a normal complete blood count, a slightly elevated serum creatinine level of 143 *μ*mol/L, and a GGT level threefold elevated. Echocardiogram obtained on admission demonstrated concentric left ventricular hypertrophy, aortic stenosis, and moderate mitral regurgitation accompanied by dilated left atrium and impaired left ventricular systolic function with ejection fraction of 39%.

**Figure 1:**
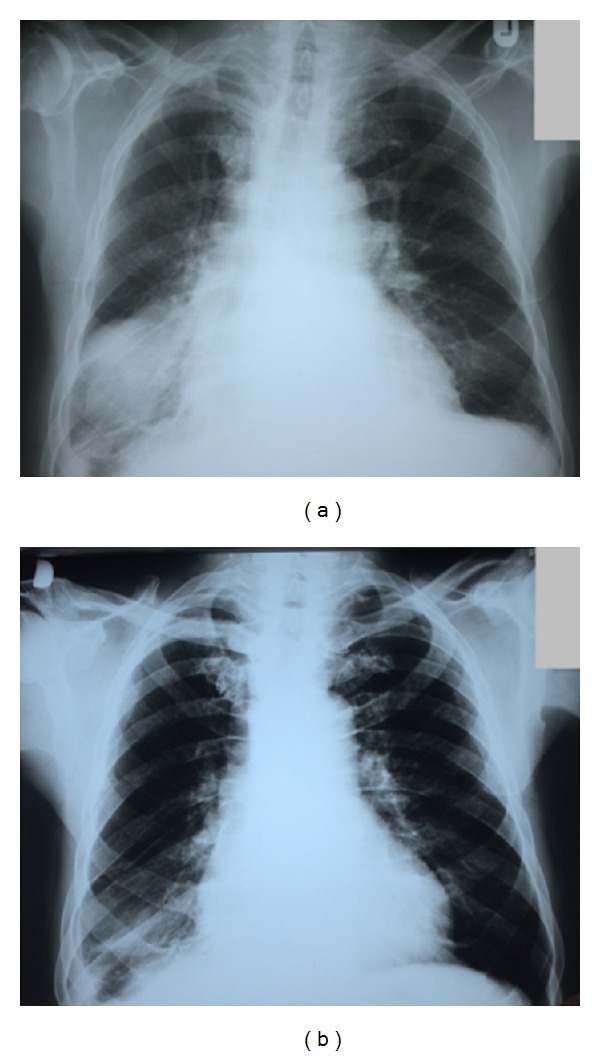
Phantom lung tumor. (a) Chest-X-ray showing homogeneous, sharply demarked opacification (phantom lung tumor) of the right inferior lobe. (b) CXR 3 days later, with absorption of the pseudotumor.

**Figure 2:**
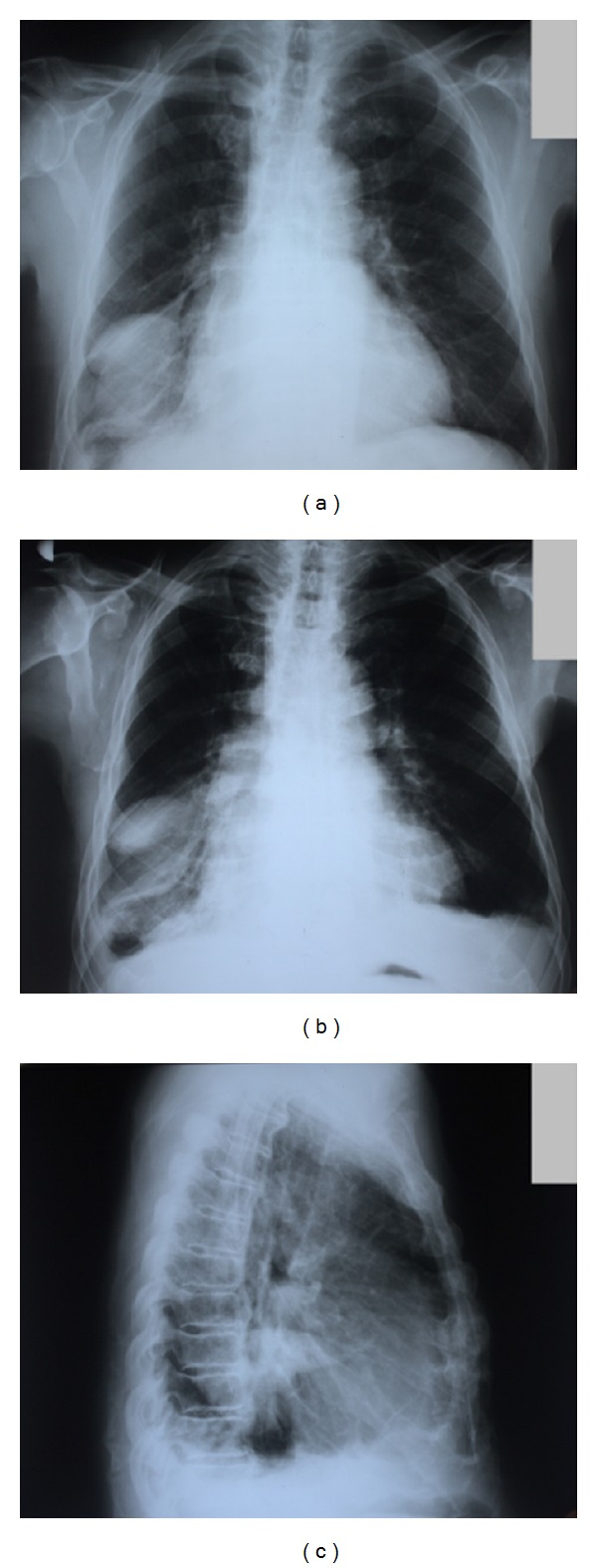
Archive chest-X-ray. (a) Chest-X-ray examinations undergone 4 years prior to admission showed similar tumor-like shadows in the same region of the right lobe. (b) Posteroanterior CXR undergone 1 year prior to admission demonstrating similar homogeneous shadows in the region of the right lower lung. (c) Lateral CXR undergone concurrently 1 year prior to admission demonstrating well demarcated opacity in the posterior aspect of the chest.

CXR showed massive sharply demarked round/oval homogeneous dense shadow 10 × 7 cm in size in the right inferior lobe (Figure 1(a)). Archive CXR undergone 1 and 4 years ago revealed intermittent appearance of the similar tumor-like shadows in the same region of the right lobe (Figure 2). Pulmonary ultrasound examination confirmed collection of the pleural fluid within oblique interlobar fissure of the right pulmonary lobe. The parenteral diuretic therapy was immediately initiated. Due to clinical and subjective improvement during the observation period, the patient was dismissed for a home treatment with a recommendation to raise peroral diuretic therapy with reduction of fluid intake. Three days later, a control CXR revealed complete resolution (Figure 1(b)) of the observed round/oval tumor-like image.

## 3. Discussion

Localized interlobar effusions in congestive heart failure (phantom or vanishing lung tumor/s) is/are uncommon but well known entities [1–3]. Due to the small number of reported cases, the incidence is difficult to estimate. In 1928, Stewart was the first one to report this entity as “interlobar hydrothorax” [4]. Phantom tumors predominantly occur in men in the right hemithorax, with three-quarters of the reported cases within the right transverse fissure and less frequently within the oblique fissure. Simultaneous occurrences in both fissures were reported in about one-fifth of cases while in the left hemithorax were described only sporadically [5]. A key role in their pathogenesis, as assumed, is related to adhesions and obliteration of the pleural space around the edge of the fissure due to pleuritis. In such setting, phantom tumors arise whenever the transudation from the pulmonary vascular space exceeds resorptive ability of the pleural lymphatics. However, this atypical intrafissural distribution of pleural effusions can also be explained by local increase in elastic recoil by adjacent, partially atelectatic lung that yields a “suction cup” effect and favors loculation of liquid even in the absence of pleural adhesions [6, 7]. The right-sided predilection of phantom tumor is best explained by the greater hydrostatic pressure existing on this side in comparison with left in congestive heart failure which results in impaired venous and lymphatic drainage causing loculation of fluid [8]. The differential diagnosis of loculated pleural effusions within the fissure includes the following: transudates due to the left ventricular failure or renal failure, exudates (parapneumonic pleural effusions, malignant pleural effusions, and benign asbestos-related pleural effusions), and hemothorax, chylothorax, and fibrous tumors originating from the visceral pleura of the interlobar fissure [9].

The presented case experienced an acute exacerbation of congestive heart failure. Characteristic posteroanterior radiographic phantom lung tumor finding was discovered: right-sided, well delineated pulmonary mass with smooth margins. Noninvasive, nonionizing pulmonary ultrasound was helpful in setting the diagnosis. Rapid resolution of the pseudotumor after management of the left heart failure provided additional evidence for the diagnosis. Several archive patient's CXRs revealed intermittent appearance of the similar tumor-like shadows in the same region of the right lobe during an acute exacerbation of congestive heart failure confirming that phantom tumors can recur during episodes of cardiac decompensation (Figure 2).

## 4. Conclusion

This case confirms efficacy of the conservative medical treatment (loop diuretics and restricted fluid intake) of the localized interlobar effusion in congestive heart failure. The possibility of phantom lung tumor should be considered and excluded in any patient presenting with congestive heart failure and an apparent lung mass on a CXR. Finally, it is necessary to highlight the importance of recognizing this condition in order to avoid unnecessary, expensive, and possibly harmful diagnostic and treatment errors.

## References

[1] Gefter WI, Boucot KR, Marshall EW (1950). Localized interlobar effusion in congestive heart failure; vanishing tumor of the lung. *Circulation*.

[2] Millard CE (1971). Vanishing of phantom tumor of the lung; localized interlobar effusion in congestive heart failure. *Chest*.

[3] Oliveira E, Manuel P, Alexandre J, Girão F (2012). Phantom tumour of the lung. *The Lancet*.

[4] Bedford DE, Lovibond JL (1941). Hydrothorax in heart failure. *British Heart Journal*.

[5] Buch KP, Morehead RS (2000). Multiple left-sided vanishing tumors. *Chest*.

[6] Fleischner FG (1963). Atypical arrangement of free pleural effusion. *Radiologic Clinics of North America*.

[7] Stark P, Leung A (1996). Effects of lobar atelectasis on the distribution of pleural effusion and pneumothorax. *Journal of Thoracic Imaging*.

[8] Rabinowitz JG, Kongtawng T (1978). Loculated interlobar air-fluid collection in congestive heart failure. *Chest*.

[9] Haus BM, Stark P, Shofer SL, Kuschner WG (2003). Massive pulmonary pseudotumor. *Chest*.

